# From Poison to Promise: The Evolution of Tetrodotoxin and Its Potential as a Therapeutic

**DOI:** 10.3390/toxins13080517

**Published:** 2021-07-24

**Authors:** Gary M. Bucciarelli, Maren Lechner, Audrey Fontes, Lee B. Kats, Heather L. Eisthen, H. Bradley Shaffer

**Affiliations:** 1 Department of Ecology and Evolutionary Biology & UCLA La Kretz Center for California Conservation Science, Institute of the Environment and Sustainability, University of California, Los Angeles, CA 90095, USA; mmlechner19@gmail.com (M.L.); brad.shaffer@ucla.edu (H.B.S.); 2 Natural Science Division, Pepperdine University, Malibu, CA 90263, USA; audrey.fontes@pepperdine.edu (A.F.); lee.kats@pepperdine.edu (L.B.K.); 3 Department of Integrative Biology, Michigan State University, East Lansing, MI 48824, USA; eisthen@msu.edu

**Keywords:** tetrodotoxin, TTX, neurotoxin, sodium channel, analgesic, therapeutic, evolutionary medicine

## Abstract

Tetrodotoxin (TTX) is a potent neurotoxin that was first identified in pufferfish but has since been isolated from an array of taxa that host TTX-producing bacteria. However, determining its origin, ecosystem roles, and biomedical applications has challenged researchers for decades. Recognized as a poison and for its lethal effects on humans when ingested, TTX is primarily a powerful sodium channel inhibitor that targets voltage-gated sodium channels, including six of the nine mammalian isoforms. Although lethal doses for humans range from 1.5–2.0 mg TTX (blood level 9 ng/mL), when it is administered at levels far below LD_50_, TTX exhibits therapeutic properties, especially to treat cancer-related pain, neuropathic pain, and visceral pain. Furthermore, TTX can potentially treat a variety of medical ailments, including heroin and cocaine withdrawal symptoms, spinal cord injuries, brain trauma, and some kinds of tumors. Here, we (i) describe the perplexing evolution and ecology of tetrodotoxin, (ii) review its mechanisms and modes of action, and (iii) offer an overview of the numerous ways it may be applied as a therapeutic. There is much to be explored in these three areas, and we offer ideas for future research that combine evolutionary biology with therapeutics. The TTX system holds great promise as a therapeutic and understanding the origin and chemical ecology of TTX as a poison will only improve its general benefit to humanity.

## 1. Overview: The Evolution and Ecology of Tetrodotoxin and Its Relevance to Medicine

**Figure 1:**
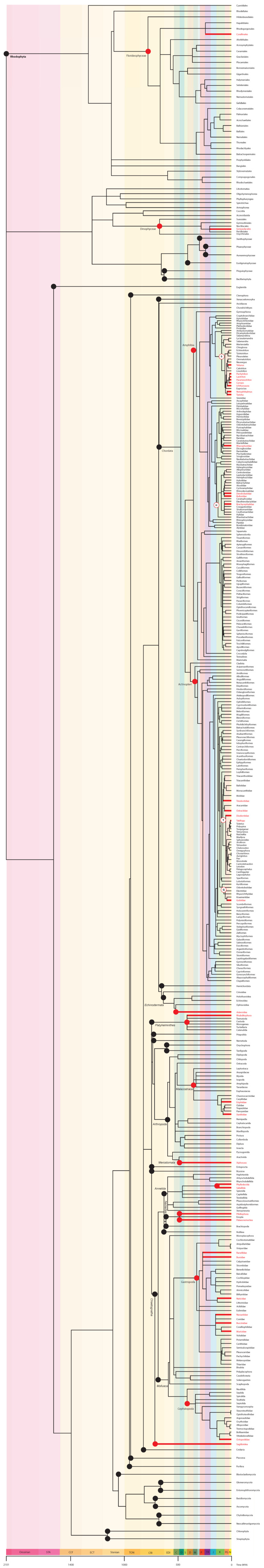
Diversity of TTX-bearing taxa throughout the eukaryote tree of life. The phylogeny shows phyla (black nodes) and descendent classes (red nodes) that contain orders, families, or genera with known TTX-bearing descendants. From top to bottom: Phylum Rhodophyta, class Florideophyceae with a single order (Corrallinales) containing at least 15 genera with one known TTX genus (*Jania*); class Dinophyceae (phylum unclassified), the order Gonyaulacales contains the only known tetrodotoxic species *Alexandrium tamarense*; Phylum Chordata, class Amphibia, there are 2 of 3 orders that consist of TTX-bearing lineages. This includes the order Anura (49 families, 3 of 4 in the superfamily Hyloidea (H) that include tetrodotoxic descendants: Brachycecephalidae, Dendrobatidae, and Bufonidae; the fourth Rhacophoridae) and the order Caudata (10 families depicted by the (S), Salamandridae include tetrodotoxic descendants). The chordates of class Actinopteri (fish) include 64 orders, but TTX-bearing species are only found in two orders, Tetraodontiformes (10 families depicted by (T), of which 4 include tetrodotoxic descendants; Triodontidae, Ostraciidae, Diodontidae, Tetraodontidae) and Gobiiformes (5 families depicted by (G), Gobiidae is the only family with descendants that possess TTX); Phylum Echinodermata, class Asteroidea (sea stars) contains over 800 species, but only two species (order Paxillosida, *Astropecten latespinosus*, *A. polyacanthus*) in a single order are known to be tetrodotoxic; Phylum Platyhelminthes, class Rhabditophora (flatworms) consists of 6 recognized classes with 2 of 8 orders (Polycladida and Tricaladida) that contain tetrodotoxic lineages in the Planocera and Bipalium genera; Phylum Arthropoda, 15 classes, but only Malacostraca (order Decapoda, family Eriphiidae and family Xanthidae, genera Demania, Atergatopsis, Lophozozymus, Zosimus) and Merostomata (order Xiphosura, family Limulidae, genera *Carcinoscorpius* and *Tachypleus*) include TTX-bearing descendants; Phylum Annelida, the class Polychaeta includes the orders Phyllodocida and Sabellida with known tetrodotoxic descendants; Phylum Nemertea, class Pilidiophora (family Cerebratulidae, genus *Cerebratulus lacteus*), and class Palaeonemertea (family Cephalothricidae, *Cephalothrix linearis*) are the only known TTX-bearing descendants; Phylum Mollusca, includes class Gastropoda that contains at least 9 tetrodotoxic species from 6 different families, including Naticidae (*Polinices didyma*), Bursidae (*Tutufa lissostoma*), Buccinidae (*Babylonia japonica*), Ranellidae (*Charonia sauliae*), Muricidae (*Rapana rapiformis, R. venosa*), and Nassariidae (*Niotha clathrata, Nassarius siquijorensis, N. semiplicatus*), but also class Cephalopoda with 7 orders and only Octopoda that includes a tetrodotoxin-bearing descendant (*Hapalochlaena maculosa*); Phylum Chaetognatha, contains tetrodotoxic descendants in two orders, Aphragmophora, with at least 3 species in 3 different genera (*Flaccisagitta enflata*, *Parasagitta elegans*, *Zonosagitta nagae*) and Phragmophora with only a single described tetrodotoxic species (*Eukrohnia hamata*). The tree is based on various subtrees produced using TimeTree [3] with molecular time estimates from Hedges et al. [4]. The final tree was manually assembled using Adobe Illustrator.

The neurotoxin tetrodotoxin (TTX) was initially discovered in pufferfish (family *Tetraodontidae*) around the end of the 19th century [1]. Subsequent research has identified the toxin in 13 phyla comprising two major domains of life (*Eukarya* and *Bacteria*), including marine and terrestrial eukaryotes such as goby fish (class *Actinopteri*, order *Gobiiformes*), octopus (class *Cephalopoda*, order *Octopoda*), gastropods (class *Gastropoda,* orders *Littorinimorpha* and *Neogastropoda*), sea stars (class *Asteroidea*, order *Paxillosida*), crabs (class *Malacostraca*, order *Decapoda*; class *Merostomata*, order *Xiphosura*), worms (class *Rhabditophora*, orders *Polycladida* and *Tricladida*) and frogs and newts (Class *Amphibia*, orders *Anura* and *Caudata*) [2] (Figure 1).

**Figure 2:**
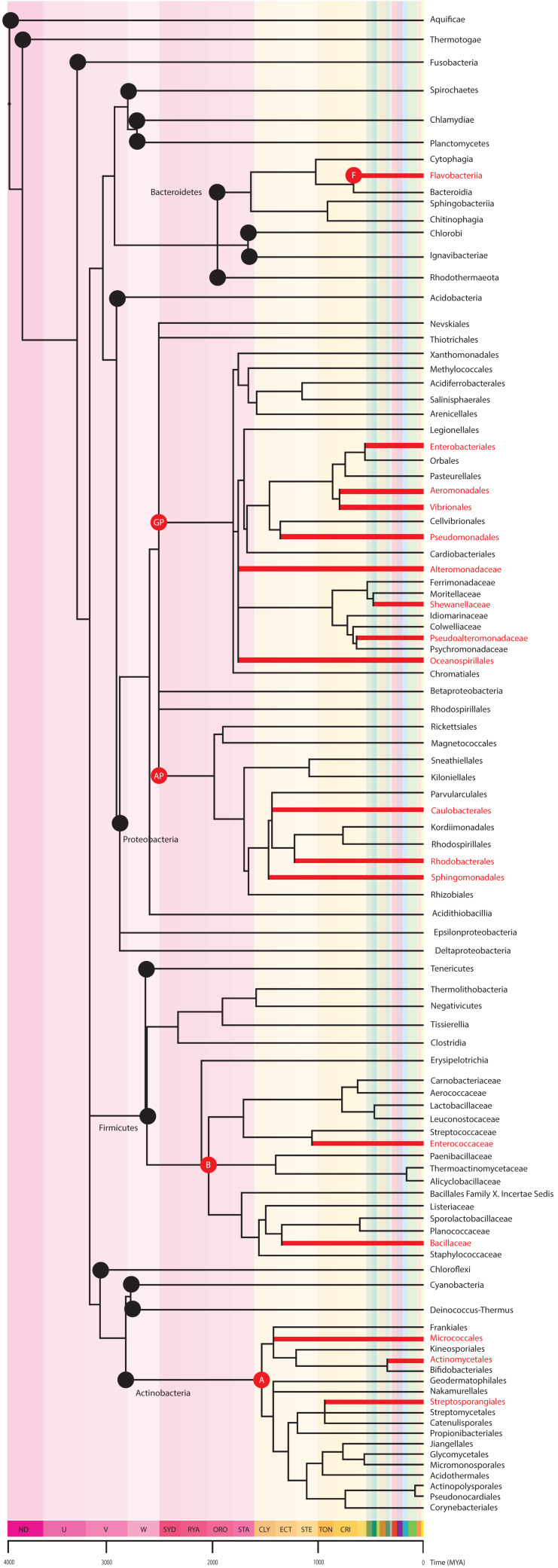
The bacteria tree of life and known TTX-producers. The phylogeny shows phyla (black nodes) and descendent classes (red nodes) that contain orders, families, or genera with known TTX-producing descendants. Of the 18 phyla, only 4 contain bacteria that produce TTX. From top to bottom, the TTX producing phyla are: Phylum Bacteroidetes, class Flavobacteriia (F) contains the order Flavobacteriales, family Flavobacteriaceae with two genera known to produce TTX (*Tenacibaculum* spp., *Flavobacterium* spp.); Phylum Procteobacteria, Class Gammaproteobacteria (GP) and class Alphaproteobacteria (AP) contain the greatest number of TTX-producing bacteria, in the orders Enterobacterales (family Enterobacteriaceae, *Raoutella* spp.; family Yersiniaceae, *Serratia marcescens*; family Pseudoalteromonadaceae, *Pseudoalteromonas* spp.; Morganellaceae, *Providencia* spp.; and the unranked *Plesiomonas* spp.), Aeromonadales (family Aeromonadaceae, *Aeromonas* spp.), Vibrionales (family Vibrionaceae, *Vibrio* spp.; *Photobacterium* spp.), Pseudomonadales (family Moraxellaceae, *Acinetobacter spp, Moraxella* spp.; Pseudomonadaceae, *Pseudomonas* spp.), Alteromonadales (family Shewanellaceae, *Shewanella putrefaciens*; family Alteromonadaceae, *Alteromonas* spp.), Oceanospirillales (family Oceanospirillacea, *Marinomonas spp),* Caulobacterales (family Caulobacteraceae, *Caulobacter* spp.), Rhodobacterales (family Rhodobacteraceae, *Roseobacter* spp.) and Sphingomonadales (family Sphingomonadaceae, *Sphinogmona* spp.); Phylum Firmicutes containing six classes but only 1 (Bacilli, B) with descendants in two orders, Lactobacillales (family Enterococcaceae, *Enterococcus* spp.) and Bacillales (family Bacillaceae, *Bacillus* spp., *Lysinibacillus* spp.) known to produce TTX; and Phylum Actinobacteria, class Actinobacteria (A) with the orders Actinomycetales (unranked *Actinomycete* spp.), Streptosporangiales (family Nocardiopsaceae, *Nocardiopsis dassonvillei*), Micrococcales (family Microbacteriaceae, *Microbacterium arabinogalactanolyticum*; family Kytococcaceae, *Kytococcus* spp.; family Cellulomonadaceae, *Cellulomonas*), Streptomycetales (family Streptomycetaceae, *Streptomyces* spp.), and Micrococcales (family Micrococcaceae, *Micrococcus* spp.). The figure was created using methods explained in Figure 1.

Overwhelmingly, it appears that TTX-bearing eukaryotes have developed associations with TTX-producing bacterial symbionts (Figure 2), but how and when tetrodotoxin production evolved is not fully resolved. It is important to note that no endogenous biosynthetic pathway for tetrodotoxin is known to occur in any eukaryote. Lineages of bacteria with descendants known to produce TTX arose as early as the Meso-Proterozoic era, dating back 1.6–1.0 billion years ago. The proximate mechanisms bacteria employ to produce TTX are also unknown. As a result, it remains unclear whether bacterial species within the 5 classes known to produce TTX all utilize similar pathways or whether each lineage independently evolved distinctly different mechanisms.

Tetrodotoxin is generally recognized as functioning as a chemical defense, but as with all traits, modification by natural selection can diversify and multiply its adaptive uses. We now recognize the unique ecological processes facilitated directly by TTX that go substantially beyond that of a poisoning agent. Toxins often play a role in animal decision-making related to foraging, and ultimately those decisions are shaped by previous exposure to the toxin. This is quite common for a number of molecules that span a spectrum from distasteful to toxic. There are far fewer examples of toxins directly mediating other behaviors. However, TTX has also been shown to affect reproduction and site fidelity [5,6,7].

Behavioral responses to TTX fall into three broad categories. Some animals show attraction to TTX, others avoid or retreat from TTX, and some respond to TTX by slowing or reducing their activity. Matsumura [6] demonstrated that TTX serves as a mating pheromone in pufferfish (*Takifugu niphobles*) attracting males to ovulating females [6]. In a separate study, parasitic copepods were shown to be more attracted to pufferfish hosts with TTX than to hosts without TTX [8] possibly because the copepods also contained TTX and their ability to find hosts depend on detecting TTX from pufferfish. Similarly, toxic snails were attracted to food that contained TTX, whereas species of non-toxic snails were not attracted to food that contained TTX [5]. California newts (*Taricha torosa*) are generally considered to use TTX as a defensive chemical, yet larvae increase hiding in response to chemical cues emitted by cannibalistic adults [9]. Subsequent electrophysiological experiments demonstrated that larvae increased hiding when exposed to TTX, which stimulated the olfactory epithelium. Further research using capture-mark-recapture data showed that TTX may also play a role in adult breeding behavior. Bucciarelli et al. [7] found that adult males generally exhibit strong breeding site fidelity, but will abandon those sites when their TTX concentrations are lower than that of other males in the breeding population. In a separate study, the results of laboratory experiments showed that invasive aquatic snails (New Zealand mud snails, *Potamopyrgus antipodarum*) also moved upstream less and retreated downstream from enclosed California newts emitting TTX [10]. Finally, Bucciarelli and Kats [11] found that waterborne TTX slowed the strike and angular velocities of dragonfly nymphs and demonstrated in field experiments that benthic stream invertebrates avoided enclosed newts, but did not avoid a non-tetrodotoxic tree frog (*Pseudacris regilla*).

In a parallel, biomedical research track, TTX has been increasingly studied as a potential therapeutic drug for various human maladies [12]. We argue that the use of TTX as a therapeutic could be improved with a clearer understanding of the origin and ecological roles of TTX. Why is understanding the ecology and evolutionary history of tetrodotoxin relevant to its potential as a therapeutic? Our view of evolutionary biologists working at this interface is that a deep understanding of the conditions by which a system functions naturally provides critical information on the limits and potential of its production and use as a pharmaceutical. As an example, venoms produced by snakes in the families Viperidae and Elapidae are widely recognized as a major source of therapeutics [13,14]. In these snakes, many proximate mechanisms responsible for their production are identified, including putative genes that produce the myriad proteins in snake venom. This understanding made it possible to evaluate how genes are being acted on by evolutionary forces (selection, mutation, drift, and gene flow; [15,16]). It is also now clear when venom production evolved and how it diversified across reptilian taxa [17,18], which allows for the targeting of distinct molecules in specific lineages. More importantly, these studies have demonstrated that toxin composition varies geographically within viperid species, and evolutionary forces differentially acting on genes that can produce drastically different combinations of venom proteins as regional variants. This has had significant consequences in the development of therapeutics, as well as antivenins [19].

We lack a comparable level of understanding of the ecology and evolution of TTX. Because its origin remains unresolved, we have no clear picture of how it evolved within the disparate classes, orders, families, and genera that possess TTX. In many instances, sister taxa of tetrodotoxic lineages have yet to be assessed for toxicity. Therefore, the trait may be more widespread, not only within clades that include tetrodotoxic lineages but also throughout eukaryotes and bacteria more broadly. As such, novel congeners of TTX with therapeutic capacity likely exist and can be explored in these unidentified lineages. Geographic variation of TTX “profiles” is likely to occur within species as a result of different coevolved selection regimes that result in unique combinations of TTX analogues with unusual binding affinities to sodium channels that may offer new therapeutic options. It would also be beneficial to determine whether all bacterial lineages produce TTX through similar or different pathways. This could be critical to achieving greater efficiency and yields when synthesizing TTX and possibly its analogues. A deeper understanding of the biology of TTX will facilitate the development of TTX-based therapeutics with greater efficacy or innovative treatments.

## 2. Mechanisms of Tetrodotoxin Poisoning and Evolution of Resistance

TTX is extremely efficacious as a poisoning agent due to its cellular targeting and mode of action [1,20], which effectively blocks action potentials in most neurons and muscle cells [20]. TTX is active in nanomolar concentrations, it is the most potent non-peptide neurotoxin known [21,22], and there are no known antidotes to TTX poisoning [23].

TTX blocks the influx of sodium through voltage-gated sodium channels (Na_V_s; see the Table 1 for commonly used abbreviations in this paper), which underlie the initiation and propagation of action potentials in almost all neurons and muscle cells [1,24]. Without sodium influx, the neurons and associated muscles are unable to function, causing paralysis [22]. A distinguishing feature of tetrodotoxin poisoning is rapid and progressive muscular weakness [25].

**Figure 3:**
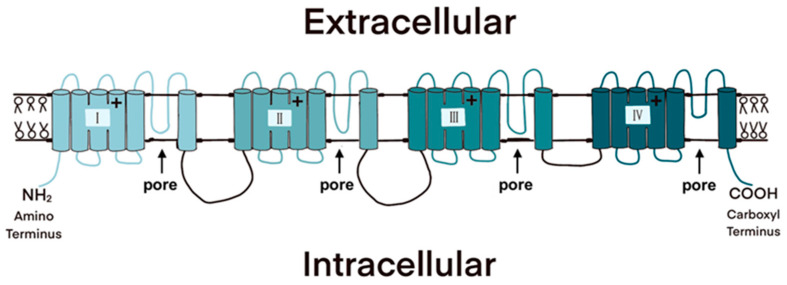
The four domains of the voltage-gated sodium channel (Na_V_) each consist of six membrane-spanning segments. The pores through which sodium ions flow are formed when the protein wraps around on itself; the extracellular loops between the fifth and sixth transmembrane segment in each domain are responsible for ion selectivity and are bound by the TTX molecule.

The Na_V_ is a membrane-bound protein that consists of a large alpha subunit flanked by two beta subunits (Figure 3). The alpha subunit comprises four domains, each of which contains six membrane-spanning segments [26]. The transmembrane segments are connected via short intracellular and extracellular peptide loops, and larger intracellular loops connect the domains. The alpha subunit is responsible for voltage sensitivity and ion selectivity and forms a central pore through which sodium ions flow into the cell. The beta subunits modulate the kinetics of the channel gate and voltage dependency and play a role in cell adhesion, signal transduction, and membrane expression [27,28]. Because they are responsible for sodium conductance and are the site of TTX binding, we will focus on alpha subunits in the remainder of this review.

The earliest vertebrates likely possessed a single *scn*, the gene that encodes the alpha subunit of Na_V_s, and the repertoire expanded to four after early vertebrates underwent two rounds of whole-genome duplication. Some of these genes were further duplicated in particular lineages. Thus, different groups of vertebrates possess multiple Na_V_s with distinct physiological properties and expression patterns [29]. Mammals possess nine voltage-gated sodium channel paralogs, designated Na_V_1.1–Na_V_1.9; a tenth member of the family, Na_x_, has lost the voltage sensor and instead functions in salt balance and fluid homeostasis [30]. In mammals, Na_V_ 1.1–1.3 and 1.6 are primarily expressed in the central nervous system (CNS), whereas Na_V_ 1.7–1.9 are expressed mainly in the peripheral nervous system (PNS). Na_V_ 1.4 and 1.5 are expressed in skeletal and cardiac muscles, respectively [31].

TTX has a high and selective affinity for Na_V_s. Negatively charged amino acids in the outer vestibule of the channel pore attract the positively-charged guanidinium group in the TTX molecule, which then binds to amino acids in extracellular loops flanking the pore (“pore loops”), blocking the flow of sodium [32]. Three of the mammalian Na_V_s, Na_V_1.5, 1.8, and 1.9, are largely insensitive to TTX. Because TTX binds to the part of the channel responsible for ion selectivity, resistance-conferring mutations generally alter ion selectivity and permeability [33]. Indeed, Na_V_1.5 which is expressed in cardiac muscle across vertebrates is much more permeable to calcium than are other Na_V_s, and its resistance to TTX is likely a pleiotropic effect of selection for enhanced calcium permeability [34,35].

TTX-bearing organisms prevent autotoxicity through two mechanisms. First, mutations in Na_V_s that affect the pore loops can prevent toxin binding, reducing or eliminating blockage of the pore. For example, the replacement of an aromatic with a non-aromatic amino acid chain in the pore loop of domain I in Na_V_1.4 reduces TTX binding in the pufferfish *Fugu pardalis* [36,37]. Among pufferfishes, identical mutations have arisen at least four times independently across the entire Na_V_ gene family, likely because only a subset of possible mutations can confer TTX resistance while still allowing for suitable channel function [33]. Rough-skinned newts (*Taricha granulosa*), which possess the highest TTX levels of any known organism, possess resistance-conferring mutations in all six Na_V_ genes [38,39]. An evolutionary analysis of the mutations in Na_V_1.6, the most widely expressed Na_V_ in the CNS, suggests that mutations in the pore loops of domains III and IV that confer slight resistance arose before a mutation in the pore loop of domain I that confers almost infinite resistance to TTX, again emphasizing the delicate balance involved in the evolution of TTX resistance [38]. In addition to their presence in TTX-bearing animals, TTX-resistant Na_V_s also occur in some animals that consume tetrodotoxic prey [40,41,42].

A second mechanism by which TTX-bearing animals prevent autotoxicity is the production of TTX-binding proteins. These unique proteins allow an organism to consume, ingest, process, and potentially store tetrodotoxin. TTX-binding proteins have only been identified in a handful of taxa, including the shore crab (*Hemigrapsus sanguineus*) [43], two pufferfishes (*Takifugu niphobles*, *Fugu pardalis*) [37,44], electric eels (*Electrophorus electricus*) [45], and five species of gastropods (*Polinices didyma, Natica lineata, Olivaminiacea, O. Mustelina*, *and O. hirasei*) [46].

A better understanding of the evolution and ecology of Na_V_s and TTX binding proteins in TTX-bearing organisms, TTX-producing bacteria, and TTX-consuming organisms will undoubtedly increase the therapeutic potential of TTX. This approach might include determining how evolutionary forces have acted on eukaryotic sodium channel mechanisms and dynamics and whether those pressures resulted in similar and shared genetic and physiological responses across taxa. In addition, learning how resistance and autoresistance have evolved may offer insight as to how best to target and deliver TTX as a therapeutic, while minimizing its deleterious side effects. Each of these questions, if answered, will provide crucial next steps in the march to improving our knowledge and use of TTX.

## 3. Effects of and Treatments for TTX Poisoning

Tetrodotoxin poisoning in humans was first documented in 1774 [47] and the overwhelming number of cases resulted from consuming pufferfish (*Fugu spp.*). Death in humans can arise from ingestion of 1.5–2.0 mg of TTX or a blood level of 9 ng TTX /mL [22] but verifying TTX poisoning can be challenging because not all TTX isomers are commercially available, precluding the development of rapid and robust assays [48]. Recent research using human-induced pluripotent stem cells has the potential to greatly enhance our understanding of lethal doses of TTX and its analogues in humans [49].

TTX spreads rapidly through the body via the circulatory system, can infiltrate the cerebrospinal fluid, and is excreted in urine [50,51,52]. TTX can cause muscle paralysis, affecting skeletal, heart, and smooth muscles; critically, paralysis of the diaphragm can lead to respiratory and cardiac failure [2,53,54]. Studies of patients affected by TTX toxicity demonstrate a higher threshold for action potential generation, slower nerve conduction velocity, and a reduction in compound muscle action potentials compared to control groups [21,25].

The severity of TTX poisoning symptoms is dose-dependent [55] and is affected by hydration state, time since ingestion until treatment, pre-existing health issues, and the degree of toxicity of the TTX structure or analogue [56]. Clinically, the degree of poisoning is classified using a four-step grading scale. Grade 1 poisoning is associated with numbness and paresthesia and possible skin sensations of tingling, tickling, prickling, or burning, with or without gastrointestinal symptoms. Grade 2 involves lingual/facial numbness, initial motor paralysis and reduced coordination, and slurred speech. Grade 3 is characterized by general flaccid paralysis with reduced muscle tone, respiratory failure, aphonia due to impairment of the recurrent laryngeal nerve, and fixed or dilated pupils. By grade 4, the victim experiences severe respiratory failure and hypoxia, hypotension, cardiac dysrhythmias, bradycardia, and unconsciousness [57]. Although there is no antidote, odds of survival improve with gastric lavage and/or oral ingestion of activated charcoal, IV hydration, hemodialysis, and injections of neostigmine to increase activity at the neuromuscular junction [1,53,58].

## 4. Medical and Therapeutic Applications of TTX

The presence of Na_V_s throughout the nervous system and muscles, and the importance of these structures in carrying out many basic physiological functions, make TTX a potent toxin but also act as a sort of omnipotent target for therapeutics (Table 2). TTX is an especially attractive option for treatment using Na_V_s because it is the most effective sodium channel blocker, able to effectively block 6 of 9 mammalian subtypes [59]. The TTX-sensitive (TTX-S) neural subtypes, Na_V_1.1, Na_V_1.2, Na_V_1.3, Na_V_1.6, and Na_V_1.7, are well distributed throughout the CNS and PNS and are associated with the generation or propagation of pain in several models [60]. Importantly, at therapeutic doses, TTX lacks affinity for Na_V_1.5, the primary Na_V_ expressed in cardiac muscle cells (Nav1.5) [61]. These factors have all increased the interest in TTX as a therapeutic, with promising results in several fields of study.

One concern regarding the systematic application of TTX is that its therapeutic index tends to be quite low, (typically 3–5 with intravenous or intramuscular injection) due to its high toxicity [62]. The therapeutic index of a drug can be understood as the ratio of the highest non-toxic exposure to that drug versus the lowest effective exposure [63]. Typically, the higher the therapeutic index of a drug, the safer that drug is for consumption [63]. While the therapeutic index of TTX is low for intravenous or intramuscular injection, several studies have shown co-administration with vasoconstrictors, local anesthetics, or via oral pellets significantly increases TTX’s therapeutic index, in addition to its overall efficacy [62,64,65].

When administered at levels far below its LD_50_, TTX has been shown to act as an analgesic in several different pain models, including neuropathic, visceral, inflammatory, cancer-related, acute, and chronic pain [12,66]. As a neurotoxin, TTX especially shows promise in its ability to treat neuropathic pain [12]. Additionally, extensive research has gone into TTX’s ability to attenuate cancer-related pain and shows potential for treating moderate to severe cancer pain [67]. Beyond pain, TTX has also shown promise in treating a variety of ailments, including, heroin and cocaine withdrawal symptoms, spinal cord injuries, brain trauma, and tumor suppression [68,69,70,71,72,73].

### 4.1. TTX as a Therapeutic: Pain

#### 4.1.1. Cancer-Related Pain

Pain arising from cancer is one of the most critical symptoms indicating the presence of the disease, and cancer pain tends to intensify as the stage of cancer advances. More than one-third of cancer patients suffer from moderate to severe cancer pain [74]. Cancer pain has various root causes, including cancer itself, treatments such as chemotherapy, and comorbidities not related to cancer, such as constipation, immobility, and thrombophlebitis [74].

Perhaps one of the most promising fields for TTX therapy is its use as a treatment for general cancer-related pain. Cancer pain is also one of the few areas where the effects of TTX as an analgesic have been tested in both animal models and humans. Results from pre-clinical and clinical trials testing the efficacy of TTX injections in the treatment of mild to severe cancer pain show promise for the future use of TTX in a clinical setting.

A 2015 study in rats showed that local injection of TTX produced a slight but significant reduction in neuropathic pain caused by the use of the colorectal chemotherapy agent Oxaliplatin. TTX was injected either 4 or 15 days after injection of Oxaliplatin in increasing doses (0.03–1.0 µg/20 µL) every 45 min [75]. At 4 days, rats given 0.1 µg of TTX or higher showed a significant increase in nociceptive mechanical threshold, with maximum effectiveness seen at the highest dose, 1.0 µg. At 15 days, the 1.0 µg dose continued to produce a statistically significant increase in mechanical nociceptive threshold, reducing chemotherapy-induced neuropathic pain [75]. Additionally, TTX in doses of 1.0, 3.0, and 6.0 µg/kg have been shown to inhibit neuropathic pain induced by a different chemotherapy agent, Paclitaxel, with no signs of toxicity or other adverse side effects [76].

Beyond rodent models, since 2007 researchers have been examining the analgesic effects of TTX for the treatment of cancer pain in human patients [77]. The results of this research found that subcutaneous injections of 30 µg TTX twice daily over a period of four days to 165 patients produced a mean analgesic response of 56.7 days, significantly higher than the placebo (9.9 days), in a double-blind, parallel-design trial across Canada, Australia, and New Zealand [78]. In both this study and a similar trial from 2008, the most common adverse effects recorded were nausea, perioral tingling, and oral numbness [78,79]. The authors concluded that TTX may provide clinically important analgesia for patients with moderate to severe cancer pain, for whom other analgesics have been ineffective or pose serious side effects such as addiction. As a result of this research, WEX Pharmaceuticals has developed TTX as a treatment for moderate to severe cancer pain; it is currently in Phase III clinical trials, the final step before approval as a clinical drug [67]. The drug, Halneuron^TM^, is being marketed as a long-lasting, non-addictive, and safe alternative to opioid treatments for cancer pain [80].

Overall, the current data are favorable for the future use of TTX as a cancer pain treatment, given its demonstrated efficacy both in rodent models and, more importantly, its ability to provide pain relief in the absence of serious side effects in humans. Based on the results of these studies, TTX could one day serve as a viable alternative to opioid-based pain treatments.

#### 4.1.2. Neuropathic Pain

Neuropathic pain is caused by an ailment or damage to the somatosensory system, causing it to function abnormally [81,82]. A notable characteristic of neuropathic pain is a spontaneous painful sensation caused by ectopic action potentials in nociceptive pathways [83]. Importantly, damage to nerves is typically followed by the upregulation of TTX-S Na_V_ expression and currents, as well as the downregulation of TTX-resistant (TTX-R) Na_V_ expression and currents. The upregulation of these TTX-S channels may contribute to the development and maintenance of neuropathic pain [84,85]. Based on these factors, TTX has been studied extensively in its ability to treat neuropathic pain and has proven to be effective across several studies in rodent models. Across these studies, no motor incoordination, motor deficiencies, respiratory distress, or sedation was recorded. TTX is of particular interest for use as a neuropathic pain treatment, as it does not produce any of the deleterious side effects characteristic of opioid use, such as addiction, inflammation, sedation, and respiratory depression [86]. Although current work is encouraging, future work in human models must corroborate the effectiveness of TTX to treat neuropathic pain. The potential use of TTX as a treatment for diverse types of neuropathic pain is discussed below.

#### 4.1.3. Allodynia and Hyperalgesia

In a 2000 paper, Lyu et al. suggested that overexpression of TTX-S Na_V_s in dorsal root ganglia (DRG) neurons following a peripheral nerve injury plays a significant role in the generation of mechanical allodynia [87]. This hypothesis was supported in a rat model where significantly reduced allodynic behaviors were measured after low-dose TTX applications improved the mechanical threshold in an affected hind paw [87].

In a study using different rat and mice pain models [88], TTX was shown to produce a significant antinociceptive effect when treating mechanical allodynia induced by a partial sciatic nerve ligation but was not as effective as morphine. At concentrations of 3 and 6 µg/kg TTX, pain scores returned to pre-injury levels in vivo; however, morphine increased pain scores to higher than pre-injury levels. The same results were seen when the two agents were used to treat thermal hyperalgesia [88]. While not as effective as morphine, the ability of TTX to return pain to the same level as pre-injury in this model is promising and indicates that TTX is effective in attenuating both mechanical allodynia and thermal hyperalgesia.

In contrast, a more recent study utilizing male rats in a full-thickness thermal injury pain model showed that systemic injections of TTX significantly reduced mechanical allodynia and thermal hyperalgesia, but with an analgesic effect as effective as or superior to morphine [86,89]. In two behavioral tests (paw withdrawal latency and grams force to withdrawal), by the second day of administration TTX was significantly more effective than morphine in blocking thermal hyperalgesia 120 min after injection. Repeated morphine injections likely produced an analgesic tolerance, which is commonly observed in opioid studies [89]. Notably, TTX did not produce this same tolerance, which would make it an appealing option as a fast-acting analgesic for severe burn injuries if the same results are observed in human patients [89].

In a different study, TTX was able to attenuate both allodynia and hyperalgesia for up to three hours in sciatic-nerve and infraorbital-nerve ligated rats [90]. Acute and subchronic administration of TTX significantly reduced both allodynia and hyperalgesia in sciatic nerve-ligated rats but had only moderate effects in infraorbital-ligated rodents [90]. Furthermore, a study of neuropathic pain caused by the chemotherapy drug Paclitaxel found that pretreatment in mice with TTX inhibited mechanical allodynia and thermal hyperalgesia as well as thermal allodynia [76]. Higher doses (3 and 6 µg/kg) of acute subcutaneous TTX were required to suppress thermal allodynia and hyperalgesia, with mechanical allodynia suppressed at all doses tested (1, 3 and 6 µg/kg). Importantly, none of the doses produced signs of TTX-induced toxicity or motor incoordination [76].

Finally, Xie et al. [91] established a critical period in which local application of TTX post-injury permanently prevented the development of both mechanical allodynia and thermal hyperalgesia in two rat models of neuropathic pain: chronic constriction injury and spared nerve injury. The immediate perfusion of TTX into the injured nerve for a period lasting at least 3–5 days prevented the development of both mechanical allodynia and thermal hyperalgesia as well as spontaneous afferent activity. However, if started 10 days post-injury, when neuropathic pain was established, TTX did not prevent the development of these conditions [91]. This result is consistent with those of previous studies showing that TTX-induced nerve blocks that were brief or initiated several days after the initial injury only provided temporary or incomplete attenuation of pain behaviors [87,91,92]. Xie et al. hypothesize that spontaneous afferent activity following peripheral nerve injury may be the trigger for other later changes within the somatosensory system that are linked to neuropathic pain. However, this pain can be avoided by temporarily blocking activity through immediate and sustained administration of TTX [91].

#### 4.1.4. Ectopic Neural Discharges

Beyond involvement in the generation of allodynia and hyperalgesia, TTX-S Na_V_ may also be responsible for the generation of ectopic discharges and ectopic neural activity in DRG, leading to hyperexcitability of neurons in the dorsal horn of the spinal cord [87]. In the mechanical allodynia study by Lyu et al. [87], epidural application of TTX was effective in blocking ectopic discharges, while in another study [93], ectopic neuronal activity after constrictive injury to peripheral nerves was inhibited by intravenously administered TTX in a dose-dependent fashion. Moreover, pre-treatment with TTX attenuated neuropathic pain caused by chronic constriction injury of the median nerve. Interestingly, these effects may be due to the activity of Na_V_ in astrocytes rather than or in addition to neurons. In one study, TTX suppressed astrocyte activation and subsequent ectopic discharges, reducing the development of mechanical hypersensitivity [94]. These results suggest TTX-sensitive channels play a significant role in the generation of these discharges, and that TTX may be a promising therapeutic agent for the treatment of neuropathic pain following peripheral nerve damage.

#### 4.1.5. Postherpetic Neuralgia

TTX is also being explored as a treatment for another type of neuropathic pain, postherpetic neuralgia (PHN), which is caused by the reactivation of the varicella-zoster virus [62]. In a 2018 study, TTX pellets (5, 10, or 20 µg/kg) were orally administered to rats to assess effects on PHN [62]. TTX pellets dose-dependently inhibited PHN (ED_50_: 5.85 µg/kg) with a much higher therapeutic index (TI: 88.45) and efficacy than intramuscular injection (TI: 4.42). Pellets prevented both mechanical and thermal allodynia more effectively than both TTX injections and alternative treatment for neuropathy (Pregabalin), demonstrating its promise as a treatment for PHN as well as the importance of the route of administration [62].

#### 4.1.6. Inflammatory Pain

Inflammatory pain is experienced as spontaneous pain and hypersensitivity in which normally innocuous stimuli are perceived as painful [95,96]. Inflammatory pain and neuropathic pain go hand in hand because both inflammatory agents and nerve damage can elicit similar pain sensations in the body, including allodynia and hyperalgesia [95]. Following inflammation, drastic alterations take place within the somatosensory system, including phosphorylation of membrane proteins and the activation of transcription factors. These changes lead to shifts in gene expression and protein function and subsequent peripheral nerve hypersensitivity, changing the perception of normal stimuli to painful [95].

This plasticity of the somatosensory system is evolutionarily adaptive, as generation of pain near a site of inflammation can promote healing of the area more rapidly through avoidance of contact [95], and brings attention to the area so that it may be treated. However, this hypersensitivity and associated pain are uncomfortable for many patients, and therefore treatments for inflammatory pain are being sought out, including, potentially, TTX.

DRG play a role in inflammatory pain, both peripherally and at their terminals in the spinal cord [95]. Nociceptive neurons within DRG express a mix of TTX-S and TTX-R Na_V_s [97]. Nevertheless, TTX holds some promise as a therapeutic in this area. For example, one study showed that injection with the inflammatory agent carrageenan led to increased expression of two TTX-S channels (Na_V_1.3 and Na_V_1.7) and an accompanying increase in TTX-S currents [97]. A separate study supported the potential role of Na_V_1.7 in inflammatory pain, by observing that a mutation in the human gene *SCN9A*, which codes for Na_V_1.7, leads to chronic inflammation with conditions including edema, erythermalgia, redness, and bilateral pain [98]. These data are promising for the therapeutic use of TTX to treat inflammatory pain, as they imply that TTX-S channels, and not just TTX-R channels, play a role in the maintenance, and perhaps the generation, of inflammatory pain. If so, therapeutic use of TTX may relieve associated pain.

Indeed, Alguacil et al. [99] observed the antinociceptive effect of subcutaneous TTX injection in a rat carrageenan model. Rats were pre-treated with TTX (2.5 µg/kg) an hour before injection of carrageenan in the right paw. Three hours post-injection, the effects of TTX on hind paw withdrawal reaction, a test of sensitivity, and inflammatory hyperalgesia, were measured. While the effects of TTX were slight, the toxin significantly reduced hyperalgesia in the affected paw. The observed minimal decrease in pain in this study as a result of a single subcutaneous injection of TTX suggests that TTX may be an ineffective treatment for severe inflammatory pain, and may be more helpful in mild, chronic inflammatory pain conditions [99].

Subsequent research tested the efficacy of TTX in treating inflammatory muscle pain caused by carrageenan and found that 0.03–1.0 µg/20 µL TTX inhibited mechanical hyperalgesia in the affected muscle [75]. Specifically, TTX was injected after treatment with carrageenan in sequentially higher doses every 45 min, leading to a dose-dependent statistically significant increase in the mechanical nociceptive threshold. Additionally, the effects of 1.0 µg of TTX were still significant 24 h after injection, demonstrating the potential use of TTX in the treatment of inflammatory pain. Furthermore, when combined with bupivacaine, an established local anesthetic, and epinephrine, a vasoconstrictor, TTX effectively reduced carrageenan-induced hind paw hyperalgesia and local inflammatory edema in a rat model, via ipsilateral or contralateral sciatic blocks [100].

As with neuropathic pain, TTX is being investigated as an alternative to current anti-inflammatory drugs, including opioid-based treatments such as morphine. To compare the treatment efficacy of TTX to morphine, researchers examined the ability of the two compounds to attenuate inflammatory pain in both an acetic acid writhing test and a formalin test [88]. In the acetic acid writhing test, morphine was more effective in reducing the number of contractions 30 min after injection than all concentrations of TTX tested. In the formalin test, TTX did not affect the initial acute phase of pain, but the highest dose (6.0 µg/kg) produced a significant analgesic effect in the second, inflammatory, phase of pain [88]. Overall, TTX was less effective than morphine, but it rendered no deleterious side effects whereas morphine led to significant sedation [88].

The potential of TTX to treat inflammatory pain seems promising due to its demonstrated ability to attenuate pain induced by inflammatory agents, possibly by increasing the nociceptive threshold in affected cells. The presence of TTX-S Na_V_s in inflammatory pain pathways is an important discovery that opens the door for TTX to be used as an inflammatory analgesic and should be explored further to understand the role TTX may play in the treatment of inflammatory pain.

#### 4.1.7. Chronic Pain

Chronic pain can be summarized as pain that persists long after the healing of any underlying injury and which no longer serves a purpose [101]. The persistence of pain after healing represents a component of vulnerability within the evolutionarily important generation of pain and can severely impact the quality of life. The continued upregulation of hypersensitivity beyond the period of healing represents a malfunctioning system that can stem from a variety of sources, including nerve damage (neuropathic pain) or inflammation, which produces continuous stimulation of the pain pathway [101].

Current pharmacological therapies available for the treatment of chronic pain are largely ineffective, providing relief to only a fraction of patients [81]. This is likely because current analgesics do not target the source of the pain but instead target ion channels and receptors in the spinal cord and brain, seeking to modulate the transmission and processing of the pain signal [102]. Analgesics that act on the CNS can produce severe side effects such as respiratory depression, sedation, euphoria, dependence, and addiction, all of which lead to dose restrictions [101]. Because of this, therapeutic agents that act directly on nociceptors are being sought out to minimize CNS-related side effects.

Through modulating Na_V_s and blocking action potentials in nociceptors, TTX could help control the hyperexcitable state of these neurons [12] and may be highly effective in the treatment of chronic pain. Several TTX-S Na_V_s, including Na_V_1.1, Na_V_1.3, and Na_V_1.7, are tied to the generation of action potentials in nociceptors and therefore could be important targets for chronic pain treatments [60,103,104]. One such TTX-S channel, Na_V_1.1, is expressed in A*δ* fibers, myelinated DRG neurons that are implicated in mechanical nociception [105]. The susceptibility of these fibers to treatment with TTX makes the toxin a promising therapeutic. Alvarez and Levine [75] demonstrated the potential effectiveness of TTX to treat ergonomic injury pain and found that TTX provided a significant antinociceptive effect on exercise-induced mechanical hyperalgesia in gastrocnemius muscle through local injection [75]. Preclinical and clinical studies in humans have also shown long-term improvement of pain through TTX injections, with minimal adverse effects that resolved quickly through the course of treatment [12,105].

#### 4.1.8. Visceral Pain

Visceral pain is the most common form of pathological pain that originates in the internal organs and encompasses a wide variety of both acute and chronic pain conditions that are experienced by millions of patients globally [66,106]. Visceral pain is usually not localized and affects multiple organs simultaneously, making it difficult to diagnose and treat [66]. While the mechanisms behind visceral pain are still not well understood, TTX-S Na_V_s may help to regulate the generation of visceral pain.

The potential effectiveness of TTX in treating visceral pain is being explored in rodent models. In an early study by Marcil et al. [88] TTX decreased visceral pain in Swiss Webster mice without any side effects in a dose-dependent fashion in an acetic acid-induced writhing test. A more recent study by González-Cano et al. [106] examined the effect of TTX on intraperitoneal cyclophosphamide-induced cystitis and found that subcutaneous injection of TTX prevented pain-related behaviors in the mice in all models tested (1–6 µg/kg), with no motor incoordination. In the same study, TTX reversed mechanical hyperalgesia induced by capsaicin as well as pain-related behaviors induced by mustard oil. Conditional knockout of Na_V_1.7 demonstrated that this subtype was not fully responsible for visceral pain propagation and suggests that several additional TTX-S subtypes help mediate this pain pathway [106].

In a separate study [107], TTX was shown to be effective in treating interstitial cystitis/bladder pain syndrome in mice [107]. The authors found that TTX-S Na_V_s regulate the excitability of mechanosensory DRG neurons in the bladder. TTX-S Na_V_s also mediate nearly all of the bladder’s afferent response to distension, again making these channels an important target for the treatment of pain [107]. Grundy et al. found that Na_V_ 1.7 was expressed in 98% of bladder DRG neurons, suggesting an important role for this subtype although its exact role in nociceptive bladder signaling is unclear [107].

Na_V_1.1 is another promising target for TTX-based treatment of visceral pain, as Na_V_1.7 and Na_V_1.1 are the two TTX-S subtypes most commonly expressed in the PNS. Na_V_1.1 is expressed in nerves that propagate mechanical pain signals to the spinal cord [103] and a recent study demonstrated that Na_V_1.1 is involved in the development of abdominal pain associated with chronic visceral hypersensitivity (CVH). Na_V_1.1 is upregulated during CVH, and inhibiting channel function reduced mechanical pain in three different mouse models. Inhibiting Na_V_1.1 also decreased colonic nociceptor mechanical responses and normalized enhanced visceromotor response to distension without interfering with normal colonic function [103]. Although TTX was not used in this particular study to inhibit Na_V_1.1, this subtype is TTX-S, suggesting that TTX could be used in the future to help treat CVH and other colon-related pain conditions [103].

### 4.2. TTX as a Therapeutic: Brain Trauma and Spinal Cord Injury

TTX could be a prime therapeutic to prevent and treat brain damage in patients that have suffered from stroke or cardiac arrest. For example, treatment with TTX after a traumatic brain injury appears to prevent the development of post-traumatic epilepsy by reducing axonal and terminal sprouting, reducing excitatory connections that can lead to hyperexcitability of circuits within the brain [72,108,109]. Although the mechanisms are not well understood, TTX may enhance pruning through suppression of neuronal activity in the injured cortex [108]. In a rat model of neocortical post-traumatic hyperexcitability, TTX was injected subdurally to the site of injury in vivo for two weeks [108]. TTX had therapeutic effects when administered immediately for 2–3 days and with a delay of up to 3 days post-injury, indicating a critical period for prevention [108]. However, further research may be necessary to confirm that TTX did not just delay epileptogenesis until 2 weeks, and was able to prevent the development of hyperexcitability [109].

TTX was also found to prevent the irreversible cell and tissue damage that normally follow hypoxia and ischemia in two different rat models [110]. In a separate study with rats, TTX was shown to postpone anoxic depolarization, the uncontrollable depolarization of neurons during brain ischemia, and related neuronal death [111]. In these cases, the effect appears to be due to the prevention of membrane depolarization, which inhibits activation of voltage-gated calcium channels as well as the release of calcium from intracellular stores, thus averting the cytotoxic effects of high intracellular calcium concentrations [108,111]. TTX also blocks the dramatic increase in intra-axonal calcium ion levels in an in vitro axonal stretch injury paradigm using human N-Tera2 cl/D1 cells, suggesting that similar results may be achievable in humans [112]. In both this and a follow-up study [113], pre-treatment with TTX was most effective in preventing calcium influx. While post-treatment between 5–20 min after injury inhibited additional calcium ion influx, it did not prevent proteolytic damage to the region of the Na_V_s responsible for channel inactivation, creating a feed-forward loop in which the membrane remains depolarized, leading to additional calcium influx [113]. These results are promising for future therapeutic uses of TTX, particularly if similar results can be observed in vivo [113].

The loss of white matter (WM) and gray matter in spinal cord injuries (SCI) is due to both primary mechanical and secondary biochemical mechanisms [71]. Because WM consists largely of axons, loss of WM has the largest effect on the recovery of function, including locomotion [114]. Na_V_s appear to play a crucial role in the loss of WM and could be a therapeutic target in helping to aid recovery following SCI [114]. In support of this idea, experimental data suggest that the abnormal influx of sodium ions following SCI is responsible for secondary phase axonal loss [115]. Microinjection of TTX into an injury site 15 min after SCI in a rat model significantly reduced WM loss assessed 8 weeks post-injury, associated with improved recovery of coordinated hindlimb movement, hindlimb reflexes, and bladder reflexes. Later studies have corroborated these results, showing a significant reduction of WM loss at 8 weeks as well as a sparing of large-diameter axons, leading to better functional outcomes provided the injections occur very close to the time of injury [114,116]. These results strongly support the potential for use of TTX in facilitating recovery of function following spinal cord damage by preserving WM.

### 4.3. TTX as a Therapeutic: Anesthetic

Researchers have also evaluated the effectiveness of TTX as an anesthetic. Early studies on the topical application of TTX to rabbit cornea found the toxin produced a long-lasting anesthetic effect (up to 8 h) with no signs of systemic toxicity, ocular irritation, or corneal thickening, even when applied repeatedly over a period of 24 h; TTX also did not hinder epithelial healing in the affected cornea [117]. These results suggest that TTX may be a viable anesthetic for corneal procedures [118], although further research is needed to determine broader applicability.

Beyond topical anesthesia, TTX has shown to be an effective local anesthetic, especially when combined with epinephrine or bupivacaine. A toxic dose of TTX is required to produce a sciatic nerve block; however, when combined with 1.1 µM epinephrine, TTX can be used at lower concentrations and the duration of blocks increased 10-fold [119,120]. Additionally, epinephrine increased the median lethal dose of TTX by a third [119]. Co-administration of bupivacaine did not increase the medial lethal dose as much, but the systemic toxicity of TTX again decreased and the anesthetic potency increased [119].

The combination of TTX and bupivacaine with dexamethasone, an anti-inflammatory, produced long-lasting nociceptive blocks but with a narrow margin of safety [121]. However, when TTX, bupivacaine, and epinephrine were combined, the duration of the nerve block was increased approximately 3-fold compared with bupivacaine and bupivacaine-epinephrine combinations [120].

Unlike the toxic dose, the amount of TTX required to provide an effective nerve block may not increase linearly with body mass. If so, the therapeutic index for use of TTX as an anesthetic in humans may be significantly higher [64,65,122]. Additionally, combining TTX with vasoconstrictors such as epinephrine, which greatly increases the therapeutic index of TTX, may be sufficient to reduce systemic toxicity [64]. Further, excessive doses of traditional local anesthetics can cause neurologic or cardiac toxicity [123]. In contrast, TTX toxicity is usually due to diaphragmatic paralysis or respiratory failure, which can be prevented through the use of respiratory support. Taken together, these results suggest that TTX, used in combination with conventional local anesthetics and/or vasoconstrictors, may prove useful in providing prolonged anesthesia with minimal side effects.

### 4.4. TTX as a Therapeutic: Tumor Suppressor

In several cancers, TTX-S Na_V_s control multiple steps within the metastatic cascade and likely play a role in maintaining metastatic cells [73,124]. For example, overexpression of Na_V_1.7 is associated with strong metastatic potential in prostate cancer both in vitro and in vivo [125] and in colorectal cancer, overexpression of Na_V_1.1 and Na_V_1.6 is associated with colorectal cancer lymph node metastasis [126]. Given the specificity of TTX and the side effects associated with many cancer drugs, these channels are attractive therapeutic targets.

Indeed, several promising studies demonstrate the potential efficacy of TTX as a tumor suppressor. In human non-small cell lung carcinoma, the overexpression of TTX-sensitive Na_V_1.7 promotes the invasion of cancerous cells [124]. TTX was found to reduce cell invasion by 50% in a non-small cell lung carcinoma cell line (H460), while not affecting non-invasive wild-type cells. The overexpression of Na_V_1.6 is also associated with the invasion capacity of cancerous cells in cervical cancer in humans [127]. In vitro, TTX reduced invasive capacity by 20% in cervical cancer cells but did not affect metastasis or proliferation [127]. The Na_V_1.6 channels were also found to be overexpressed in the membrane and cytoplasmic compartments of cervical cancer cells, where they may play a role in the metastatic properties of the cell [127]. Thus, while some ambiguity surrounds the potential of TTX to attenuate metastasis in cervical cancer cells, the results suggest an important role of Na_V_1.6 channels in the progression of this cancer type.

Finally, TTX-S channels Na_V_1.1–1.4 and Na_V_1.7, as well as the TTX-R Na_V_1.5, are overexpressed in ovarian cancer cells [128]. Treatment with a very high dose of TTX, 30 µM, significantly reduced migration and invasion, but not proliferation, of two ovarian cancer cell lines, Caov-3 and SKOV-3, but a lower dose that was sufficient to block TTX-S channels (1 µM), did not [128]. These data suggest a link between TTX-R Na_V_1.5 and the progression of ovarian cancer, leaving open the question of whether targeting TTX-S Na_V_s would be effective in treating ovarian cancer.

In addition to these in vitro studies, a few experiments have explored the use of TTX as an anti-cancer agent in vivo. For example, two groups of Swiss albino mice were injected with TTX in addition to Ehrlich ascites carcinoma (EAC) cells. Animals administered 1/10 LD_50_ TTX showed the largest increase in days of lifespan, 46.6 ± 4.2%, and those administered 1/20 LD_50_ TTX exhibited an increase of 26.7 ± 2.6% relative to controls. Both TTX treatments also inhibited tumor cell production by 40–60%, and TTX showed an obvious cytotoxic effect on the cancer cells, causing apoptosis and a decrease in the volume of peritoneal fluid [129]. In a separate study, female mice were injected with EAC cells and treated with TTX and/or doxorubicin, a chemotherapy agent that produces serious adverse side effects, including irreversible degenerative cardiomyopathy and congestive heart failure. TTX on its own produced a stronger effect on tumor weight and survival time (days) than the other treatments and produced fewer side effects, suggesting that TTX could be preferable to some established anti-tumor agents [130]. Future research should focus on the potential long-term use of TTX as a tumor-suppression agent as well as side effects that may arise from repeated treatment. However, the overexpression of Na_V_s in several types of cancer provides an ideal target for reducing cancer progression with minimal side effects.

### 4.5. TTX as a Therapeutic: Heroin and Cocaine Addiction

Heroin withdrawal syndrome and subsequent relapse is a major issue for the successful treatment of heroin addiction. Even after long periods of abstinence, drug-associated environmental cues or exposure to stressors can induce drug use in recovering addicts [131]. Therefore, many clinicians try to prevent such relapses from occurring in the long term. Currently, prescription opiates are used to treat withdrawal symptoms in a controlled fashion but are not a long-term option due to their addictive nature [69]. In a 2009 double-blind, placebo-controlled study involving 45 individuals, a single intramuscular injection of 10 µg TTX significantly reduced cue-induced anxiety and craving in abstinent recovering heroin addicts, with no significant effect on blood pressure or heart rate [131]. In a separate double-blind, placebo-controlled trial involving 216 patients, intramuscular injection of either 5 or 10 µg TTX three times a day significantly reduced withdrawal symptoms by the third day of the trial in newly abstinent addicts with no adverse effects on respiration or blood pressure [69]. Both of these studies suggest that TTX may be effective in attenuating the symptoms of opiate withdrawal and preventing relapse. TTX may also provide an attractive alternative to opioid treatments because experimental data suggest that it is not addictive and recognized effective doses do not pose a cardiovascular or neuronal risk [78,101]. However, the mechanism through which TTX relieves withdrawal symptoms is unknown, as are other potential side effects (e.g., on blood oxygen saturation, breathing rate, or skin temperature) that were not examined in these two studies.

The mechanism through which TTX alleviates heroin withdrawal symptoms may also be similar to the pathway through which TTX suppresses cocaine-seeking behavior in laboratory rats. The basolateral amygdala and the nucleus accumbens are involved in drug-seeking behaviors in several species, including humans [132]. The nucleus accumbens is activated when cocaine is ingested, and metabolic changes in the basolateral amygdala are associated with drug craving and can be induced by drug-related stimuli [132]. In a study with Sprague-Dawley rats, microinjection of TTX was used to temporarily inactivate either the basolateral amygdala or the nucleus accumbens bilaterally to study their effect on reward-seeking behaviors [132]. When injected into the basolateral amygdala, TTX disrupted secondary reward-seeking behavior (tone and light with no cocaine) but did not affect primary reward-seeking behavior (cocaine). The opposite effect was seen when TTX was injected into the nucleus accumbens, attenuating primary reward-seeking behavior while having no effect on secondary reward-seeking behavior [132]. In a separate study, bilateral TTX injections reduced cocaine drug-craving behavior in rats after inactivation of the basolateral amygdala, anterior cingulate cortex, prelimbic cortex, or infralimbic cortex, with no overall effect on locomotor activity [70]. The authors suggest that their results support a role for the basolateral amygdala as well as the dorsomedial prefrontal cortex in upholding cocaine-induced drug-seeking behavior, which could result in a relapse in recovering addicts. The mechanism through which this suppression occurs may be similar in heroin withdrawal patients, as the basolateral amygdala has also been implicated in the reinstatement of heroin-seeking behavior in rats [133].

## 5. Concluding Thoughts

Commercially available TTX is currently harvested from marine animals, largely pufferfish, and methods of synthesis must be improved before TTX can be widely used as a therapeutic. In laboratory synthesis, yield is extremely low, returning 0.34–1.82% of product via synthesis protocols requiring 23 to 67 reactions. In addition, only a handful of the thirty known analogues have been laboratory synthesized and even fewer are commercially available [1]. Improved synthesis would facilitate research and therapeutic use of both TTX and related compounds.

Further research must test the capability of TTX to treat ailments beyond animal models to establish the safety and efficacy of these treatments in patients. In addition, as different analogues of the toxin may have different efficacies in treating various conditions, more work should be put into determining which analogue of TTX is the most effective therapeutic in each potential treatment scenario.

One of the major obstacles to TTX’s implementation as a widespread therapeutic is the uncertainty regarding the toxin’s ability to cross the blood-brain barrier. While many studies have claimed that TTX is either unable or severely limited in its ability to cross the blood-brain barrier, no direct evidence supports this claim. A review by Kao claims that experiments performed by Ogura (1958) in rats using crystalline TTX resulted in detectable amounts of TTX in the rat’s blood and brain after a single subcutaneous injection [51,134]. Furthermore, TTX has been detected at levels ranging from 0.045–4.5 µg TTX/g tissue in the brain of the rough-skinned newt (*Taricha granulosa)*, far higher than levels in the blood, indicating that the toxin may have an ability to cross this barrier [135]. Before research on TTX’s potential as a therapeutic can continue, more work should be done on understanding the toxin’s ability to penetrate the blood-brain barrier. However, if TTX does cross the blood-brain barrier, then the toxin potentially can still serve as a therapeutic, albeit potentially with modification to the molecule to limit unintended effects associated with blood brain-barrier transgression.

Another area of research that will become increasingly relevant as the therapeutic potential of TTX is evaluated is optimizing the efficacy of administration, and recent studies have begun to explore this issue. Oral TTX pellets were significantly more effective than intramuscular injection in treating postherpetic neuralgia, with a higher therapeutic index than injection [62]. In a follow-up study, enteric sustained-release TTX pellets were examined for analgesic effect in an acetic acid-induced writhing test in rats. Doses between 20 and 80 µg/kg produced an analgesic effect that lasted 1.5–9.0 h after administration, with the strongest effects seen between 3–6 h [65]. Oral administration of TTX, if effective, would greatly facilitate its use in clinical treatment due to the increase in patient compliance compared to intramuscular injections.

A review by Melnikova et al. [122] found significant improvement in the efficacy of TTX when co-administered with vasoconstrictors, local anesthetics, and chemical permeation enhancers. However, the greatest efficacy in administration was observed when TTX was encapsulated with microparticles and liposomes conjugated to gold nanorods [121,136,137]. Specifically, encapsulation with silica nanoparticles reduced systemic toxicity while increasing the duration of the anesthetic effect of TTX [136]. However, the use of micro- or nanoparticles is sub-optimal, as larger particles can hold a larger quantity of drug and are less likely to disintegrate [138].

Through its ability to selectively target TTX-S Na_V_s throughout the body, TTX has shown to be a promising therapeutic agent, with various uses across a variety of ailments. Especially within the treatment of various types of pain, TTX has shown to be an effective analgesic. However, the neurotoxin has also proven useful in several surprising areas, including tumor suppression and attenuating drug withdrawal symptoms. While continued research is necessary, many studies demonstrate the therapeutic potential of TTX. Through the same channels that TTX has evolutionarily targeted to induce pain and even death, it is also able to relieve pain and possibly protect the body from disease.

## Figures and Tables

**Table 1:** Commonly used abbreviations.

Abbreviation	Definition
CNS	Central nervous system
CVH	Chronic visceral hypersensitivity
DRG	Dorsal root ganglia
EAC	Ehrlich ascites carcinoma
LD	Lethal dose
NaV	Voltage-gated sodium channel
PHN	Postherpetic neuralgia
PNS	Peripheral nervous system
SC	Spinal cord
SCI	Spinal cord injury
TTX	Tetrodotoxin
TTX-R	Tetrodotoxin-resistant
TTX-S	Tetrodotoxin-sensitive
WM	White matter

**Table 2:** TTX-S Na_V_s related pathologies discussed in this paper.

TTX-S Na_V_ Subtype	Location(s) in Body	Associated Pathologies
Na_V_1.1	CNS Microglia PNS ^1^ Ventricular cardiomyocytes	Chronic pain Chronic visceral hypersensitivity Colorectal cancer Ovarian cancer
Na_V_1.2	CNS PNS (low expression) SC (lamina I/II) Ventricular cardiomyocytes	Ovarian cancer
Na_V_1.3	DRG (low expression) Embryonic sodium channel SC (lamina I/II—adults) Ventricular cardiomyocytes	Chronic pain Inflammatory pain Ovarian cancer
Na_V_1.4	Skeletal muscle Ventricular cardiomyocytes	Ovarian cancer
Na_V_1.6	Epidermal free nerve terminals Keratinocytes Microglia Nodes of Ranvier SC, PNS ^1^ Ventricular cardiomyocytes	Cervical cancer Colorectal cancer
Na_V_1.7	PNS (all types of DRG neurons) SC ^1^ Epidermal free nerve terminals	Chronic pain Inflammatory pain Interstitial cystitis, bladder pain syndrome Intraperitoneal cyclophosphamide-induced cystitis Mechanical hyperalgesia Non-small cell lung carcinoma Ovarian cancer Prostate cancer

^1^ TTX-S Na_V_ subtype is in DRG, myelinated Aδ fiber neurons.

## Data Availability

Phylogenies and tree data can be found at www.timetree.org, accessed on 23 July 2021.
